# Down-regulation of platelet adhesion receptors is a controlling mechanism of thrombosis, while also affecting post-transfusion efficacy of stored platelets

**DOI:** 10.1186/s12959-019-0209-5

**Published:** 2019-10-23

**Authors:** Ehteramolsadat Hosseini, Maryam Mohtashami, Mehran Ghasemzadeh

**Affiliations:** 1 Blood Transfusion Research Centre, High Institute for Research and Education in Transfusion Medicine, Iranian Blood Transfusion Organization Building, Hemmat Exp. Way, Next to the Milad Tower, PO Box: 14665-1157, Tehran, Iran; 2 Australian Center for Blood Diseases, Monash University, Melbourne, Victoria 3004 Australia

**Keywords:** α_IIb_β_3_, Adhesion, Ectodomain shedding, GPIbα, GPVI, Microparticle, Platelet refractoriness, Transfusion, Thrombosis

## Abstract

Physiologically, upon platelet activation, uncontrolled propagation of thrombosis is prevented by regulating mechanisms which affect the expression and function of either platelet adhesion receptors or integrins. Receptor ectodomain shedding is an elective mechanism which is mainly involved in down-regulation of adhesion receptors GPIbα and GPVI. Platelet integrin α_IIb_β_3_ can also be modulated with a calpain-dependent proteolytic cleavage. In addition, activating signals may induce the internalization of expressed receptors to selectively down-regulate their intensity. Alternatively, further activation of platelets is associated with microvesiculation as a none-selective mechanism which leads to the loss of membrane- bearing receptors. In a non-physiological condition, the storage of therapeutic platelets has also shown to be associated with the unwilling activation of platelets which triggers receptors down-regulation via aforementioned different mechanisms. Notably, herein the changes are time-dependent and not controllable. While the expression and shedding of pro-inflammatory molecules can induce post-transfusion adverse effects, stored-dependent loss of adhesion receptors by ectodomain shedding or microvesiculation may attenuate post-transfusion adhesive functions of platelets causing their premature clearance from circulation. In its first part, the review presented here aims to describe the mechanisms involved in down-regulation of platelet adhesion receptors. It then highlights the crucial role of ectodomain shedding and microvesiculation in the propagation of “platelet storage lesion” which may affect the post-transfusion efficacy of platelet components.

## Highlights

- Different mechanisms of internalization, microvesiculation and ectodomain shedding modulate platelet receptors
- Platelets lose the expression and function of adhesion receptors during storage
- Ectodomain shedding and microvesiculation are mainly involved in platelet storage lesion (PSL)
- Platelet transfusion can be complicated by platelet refractoriness
- PSL decreases post-transfusion efficacy of platelets

## Introduction

**Fig. 1:**
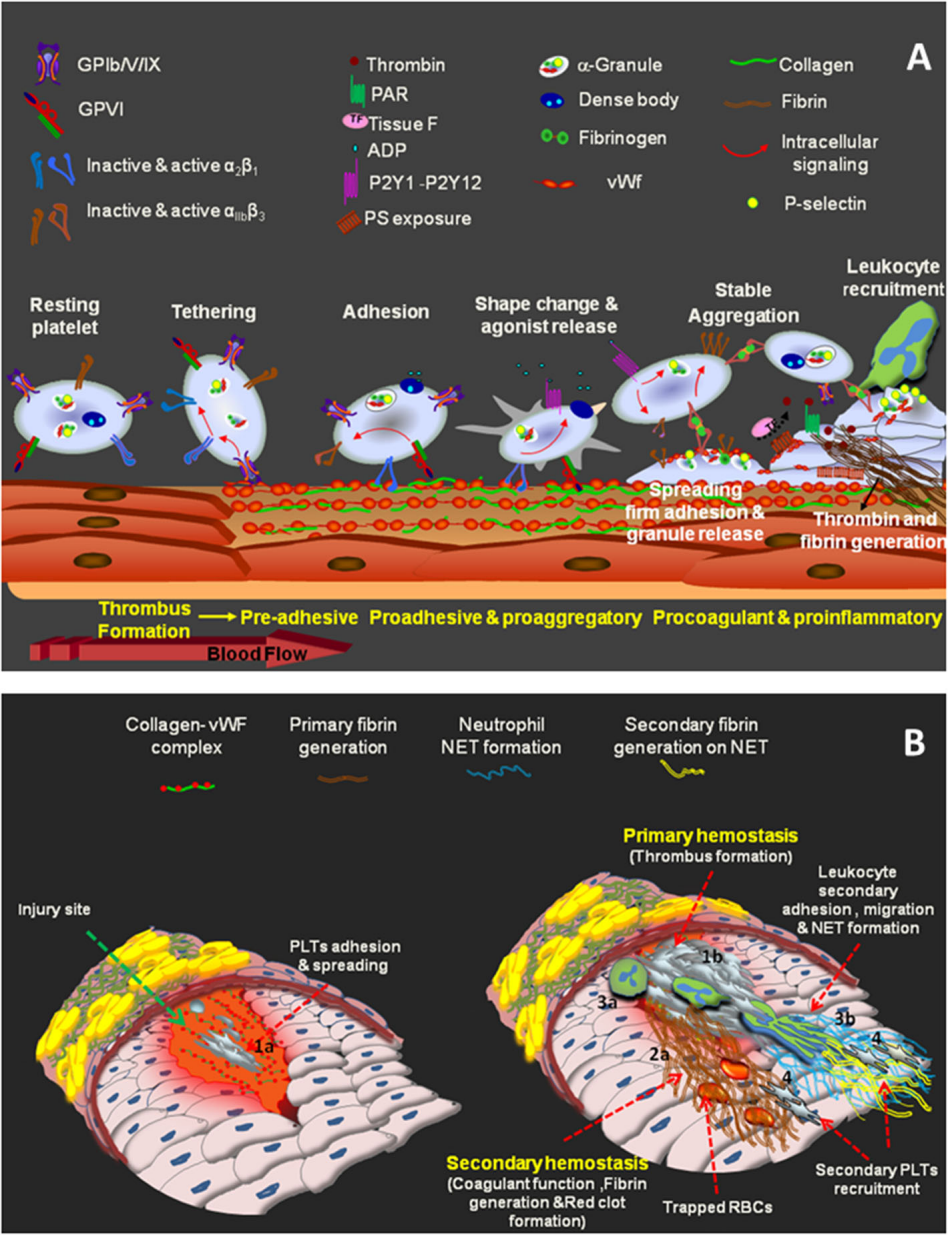
**a** Different Stages of Platelet Adhesion to the Site of Vascular Injury. Resting platelets are initially captured on the site of vascular injury via the interaction between GPIb/V/IX and immobilized vWF ***(Tethering).*** Platelets firmly adhere to the sub-endothelial matrix through the engagement of collagen receptors α2β1 and GPVI ***(Adhesion)***. This triggers potent inside out signals inducing ADP release from dense bodies *(****Shape change & agonist release****)* as well as activating platelet major integrin α_IIb_β_3_. Integrins facilitate platelet ***spreading*** and subsequent ***aggregation*** through the binding to vWF and fibrinogen. Activating signals down-stream engaged receptors induce the ***release of granule*** contents including P-selectin which provides an efficient scaffold for ***leukocyte recruitment*** linking pro-aggregatory phase of platelet activation to pro-inflammatory function. On the other hand, the accumulative signals further activate platelets and induce sustained calcium influx which results in the surface exposure of phosphatidylserine (PS) and pro-coagulant function leading to thrombin production and fibrin generation at the site of injury. Interacting with PAR receptors, generated thrombin also acts as a potent agonist which supports more efficient ***pro-inflammatory and pro-coagulant*** function. **b** Primary and secondary hemostasis: mutual links between pro-inflammatory and pro-coagulant function 1- (**a)** Followed by the injury, platelet recruitment to the exposed sub-endothelial matrix leads to the formation of a developing thrombus(**b**) which express either pro-inflammatory molecules (mainly P-selectin) or pro-coagulant phospholipids (***primary hemostasis***). **2**- Pro-coagulant matrix presented by platelets provides an efficient scaffold for fibrin generation (**a**) which supports ***secondary hemostasis*** by the conversion of the white thrombus to a red clot containing a planner of fibrin network and trapped RBCs.**3-** Platelets recruits leukocyte(**a**) while during their crosstalk, neutrophils get fully activated and release their chromatin contents as extracellular NET(**b**). The negatively charged NET materials provide an efficient pro-coagulant scaffold for fibrin generation**. 4-**Platelets may also interact with generated fibrin while creating a secondary thrombus

Platelets typically circulate in the bloodstream for 7–10 days and their principal function is to survey the inner lining of blood vessels to detect and seal any breaches in the vasculature by the creation of thrombi. The firm adhesion of platelets to the site of injury forms a monolayer which serves as a reactive site for further recruitment of free-flowing platelets. Platelet accumulation is dependent on platelet-bound Willebrand factor (vWF) and fibrinogen which serves to cross-link adjacent platelets and promote stable platelet aggregation [1, 2]. This process is essential for the formation of a primary hemostatic plug (white thrombus) and also for the development of pathological thrombi at site of atherosclerotic plaque rupture [3]. Of note, in a developing thrombus further activation converts platelets from a proaggregatory to a to pro-coagulant phenotype which enables the assembly of the coagulation reaction complexes (the Tenase and Prothrombinase complex) on the cell surface, necessary for thrombin and fibrin generation. This supports secondary hemostasis by the conversion of the white thrombus to a red clot containing a planner of fibrin network and trapped RBCs [4]. Nonetheless, physiological thrombus formation (mural thrombi) is tightly regulated to avoid excessive platelet accumulation at the injury site and vascular obstruction, the principal pathological process causing heart attacks and ischaemic stroke [5]. Figure 1 demonstrates different steps of platelet tethering, adhesion, aggregation and thrombus formation.

## The important functions of platelet adhesion receptors

Classically, thrombus formation on the site of vascular injury is triggered by the interaction of main platelets adhesive receptors Glycoprotein Ib/V/IX and Glycoprotein VI (GPVI) with their specific ligands which are exposed to circulation followed by endothelial damage. The initial capturing of free flowing platelets occurs through the binding of GPIb/V/IX to immobilized von vWF expressed at sites of vascular injury. This interaction slows down platelet movement and allows other adhesion receptors with slower-binding kinetics, including integrin α2β1 and GPVI, as the most potent adhesion receptor to be engaged with the exposed collagen in sub-endothelial matrix. Followed by GPVI ligation with collagen, the induction of strong inside-out signals induces platelet activation and release. These events are associated with integrin activation on the surface of both adhered and adjacent free flowing platelets while the interaction of these activated integrins with fibrinogen/VWF can crosslink platelets to make aggregation and thrombus formation (Fig. 1a). Integrin ligation induce potent outside-in signals which augment cytosolic calcium influx associated with the conversion of pro-aggregatory phenotype of platelets located on developing thrombi to pro-inflammatory and pro-coagulant platelets [3]. P-selectin expressing platelets recruit leukocytes to the site of vascular injury while pro-coagulant platelets provide a highly efficient scaffold for coagulation- cascade activity and fibrin generation which develop clot formation [6]. At this stage, polymerized fibrin has also shown to recruit circulating rest platelets lacking activated integrin through the interaction with adhesive receptors Glycoprotein Ibα (GPIbα) and GPVI [7]. This second phase of platelet recruitments may provide a new scaffold for thrombin generation enhancing coagulant function. Developing thrombus also expresses a verity of pro-inflammatory mediators and receptors (Fig. 1b). Leukocytes recruitment to thrombi is initiated by the interaction between P-selectin and P-selectin glycoprotein ligand-1 (PSGL-1) which respectively are expressed on activated platelets and circulating rest leukocytes. Via this interaction, leukocyte begins to tether and roll around the thrombus while being affected by chemokines and chemoattractant which are continuously released from activated platelets. This cellular crosstalk induces potent inside-out signals which activate leukocyte integrin Machrophage-1 antigen (MAC-1). Followed by MAC-1 interaction with its main ligand in platelets, GPIbα, leukocytes firmly adhere to thrombi while getting fully activated and polarized. These activated leukocytes may release chromatin neutrophil extracellular traps (NETs) which ensnare more circulating cells including platelets while providing an efficient negatively charged matrix that propagates pro-coagulant function [6, 8] (Fig. 1b).

## Different mechanisms modulating platelet adhesion receptors

Changes of platelet receptors expression by different stimuli or during storage can influence their surface density and ligand binding capacity, signal transduction as well as receptor cross-linking and clustering. Regardless of platelets functional activity which can be affected by these alterations, physiologically, the changes may also control platelet aging or clearance. Studies have shown that the expression of adhesion receptors on the surface of platelets can be modulated by four different mechanisms including A) internalization, B) microvesiculation (microparticulation), C) secretion and D) ectodomain shedding. In addition, followed by ectodomain shedding, soluble forms of receptors lacking a transmembrane domain may also interfere with receptor functional activity while during microvesiculation, microparticle -bearing receptors may also play some important physio-pathological roles [9–11]. The controlled termination of signaling pathways after ligand-induced activation is an important mechanism to ensure appropriate signal intensity and the consequent cellular response [12]. Internalization, secretion and ectodomain shedding are three mechanisms involved in dampening ligation capacity and signal transduction via the selective removal of receptors from platelet membrane [13, 14]. These mechanisms which are ignited from early stage of platelet activation modulate different aspects of cellular functions including platelets trafficking and adhesion to the site of vascular injury as well as their pro-inflammatory and pro-coagulant functions during thrombus formation. Damaged receptors specially affected by oxidant stress have also shown to be eliminated by such selective removal mechanisms [15]. On the other hand, microvesiculation is considered as a none-selective mechanism of receptor removal, usually from fully activated platelets during membrane shedding or from apoptotic platelets by membrane fragmentation. Although platelets continuously shed microparticles (MPs) even under resting condition, significant microvesiculation occurs in post-activated or apoptotic end stage platelets [16].

### Internalization of platelet receptors

Internalization can also affect platelet receptors redistribution by different specific and nonspecific mechanisms. One of these mechanisms is receptor translocation into the open canalicular system (OCS). This is an important mechanism to regulate the redistribution of GPIb and GPIV [17] . GPVI, platelet G-protein coupled receptors (PAR-1, PAFR, P_2_Y_1_, P_2_Y_12_), integrins (α_2_β_1_, α_IIb_β_3_), Platelet-derived growth factor receptor α, thrombopoietin receptors and prostacyclin receptors are also other platelet receptors which are internalized by different mechanism [18–24].

#### General mechanisms of receptor internalization

Upon activation, internalization redistributes the receptors while shuttling them from the plasma membrane to endosomes where they may experience inactivation process through lysosomal degradation [25, 26]. Generally, endocytosis is triggered by ubiquitin molecules interaction with activated receptors and their related proteins which are associated with tyrosine kinase dependent Phosphorylation [12]. One of the important components of endocytic machinery is clathrin which is likely regulated by ubiquitination processes [27]. When a clathrin-coated pit forms on the inner surface of the cell membrane, it then buds inside the cell and forms a coated vesicle containing receptors and other membrane associated molecules. Cytoplasmic proteins including dynamin and adaptors such as adaptin may be also involved in endocytosis process [28]. Other molecular factors which may affect membrane receptors redistribution are included in cytoskeletal proteins and lipid composition [29].

Receptor endocytosis may also occur constitutively without cellular activation. This is a clathrin- independent pathway of endocytosis which is not affected by receptor antagonisms or associated with receptor signaling [26]. In some condition receptor redistribution may be induced even by non-molecular factors such as external physical stress which affects membrane integrity and cause none specific endocytosis [29].

### Platelet Microvesiculation

Notably, since more than 80% of circulating microparticles express platelet antigens in healthy individuals, the physiological importance of platelet-originated microparticles and their relevance with the status of different diseases especially cardiovascular and inflammatory disorders, including arterial thrombosis, heparin-induced thrombocytopenia, immune thrombocytopenia, malaria infection, *acquired immune deficiency syndrome* (AIDS), and rheumatoid arthritis have been so far of interests in numerous researches [30, 31]. Platelets release two types of vesicles including exosomes (approximately 40–100 nm in diameter) which are shed by exocytosis from the multivesicular bodies as well as alpha-granules and microparticles (approximately 100–1000 nm in diameter) that are released by budding of the cytoplasmic membrane or by platelet fragmentation during apoptosis [10]. As a general marker, like other type of microparticles, platelet derived microparticles; (PMPs) usually express Annexin-V or tissue factor (TF). PMPs also express variety of platelet surface molecules including adhesive receptors (such as GPIbα, β_3_ integrin (GPIIIa, CD61), α_IIb_-integrin (GPIIb, CD41), GPVI, P-selectin, lysosomal-associated membrane protein3 (LAMP3, CD63), thrombospondin − 1 and Platelet endothelial cell adhesion molecule (PECAM)-1 [31, 32]. Therefore, membrane loss during microparticulation is associated with the loss of platelet surface proteins including adhesive receptors. Given this, microparticle formation can be considered as a down-regulating mechanism of platelet adhesiveness to reactive matrixes. However, PMPs show diverse phenotypes based on their activation states. Resting platelets-derived microparticles are phosphatidylserine (PS) negative, which not expressing pro-inflammatory molecules including P-selectin whereas activated platelets express PS, P-selectin and CD40 ligand (CD40L) [33]. In addition, as an another important pro-inflammatory molecule, PMPs also showed to express platelet-activating factor (PAF) [34]. Amongst adhesion molecules expressed by microparticle, GPVI also detected on megakaryocyte microparticles while not being expressed by microparticles released from activated platelets [35]. This may suggest that, contrary to other adhesion receptor GPIbα (which is significantly expressed by PMPs), GPVI down regulation on platelets cannot be affected by microparticulation and mainly modulated by other mechanisms including ectodomain shedding or internalization**.** Notably, regardless of expressed molecules, PMPs also comprise a vast array of biologic materials including growth factors, coagulation factors, enzymes, chemokines, cytokines, complement proteins, apoptosis regulators, bioactive lipids, miRNAs, β-amyloid precursor protein, Ca^2+^-dependent protease) calpain (, which may play important roles in different biological functions [36].

#### Microvesiculation mechanism in platelets

Membrane integrity in resting platelets can be affected with intensive physical stress induced by some pathological states such as the presence of abnormal shear force in atherosclerotic vessels, mechanical artificial valve and microangiopathic conditions [37]. Contrary to agonist-induced microparticle generation or what occurs in activated platelets, Destabilization of the actin cytoskeleton in resting platelets can trigger microparticle shedding by the mechanisms which are not dependent to Ca^2+^ elevation and calpain activity [30]. However, the involvement of the phosphoinositide 3-kinase signaling pathway in the articulation of α_IIb_β_3_-integrin with actin dynamics might be one of the possible mechanisms [38]. Upon platelet activation, the elevation of intracellular Ca^2+^ and ROS generation (through cyclooxygenase and NADPH oxidase) induce calpains activation and cytoskeletal changes. Clapains cleave skeleton associated proteins in platelet membrane while aberrantly destabilizing actin polymerization and cytoskeletal arrangement [39, 40]. Generally, Calpain activity in addition to the loss of membrane integrity induced by PS exposure, are the main mechanisms behind microparticle shedding in activated platelets. Moreover, shear stress-induced PMPs formation may also occur during platelet activation and adhesion through the interaction between GPIbα and vWf where the loosely adhered platelets are tethering off from the adhesive matrix. This means that the circulating detached platelets lose some parts of their membrane contents including adhesive receptors [41]. With fairly different mechanisms involved, platelet apoptosis is also associated with the significant microparticle formation during membrane fragmentation which might be mechanistically different from calcium dependent budding of cell membrane in activated platelets. Platelet apoptosis is associated with mitochondrial damage, cytochrome release and caspase activity which leads to both calpain dependent and independent cytoskeleton cleavage/reorganization resulting in microparticles release from remnant of the apoptotic cell [16, 42]. Activation based receptor modulation has been also shown to be relevant with microparticle formation. In addition to calpain, GPIbα modulation during platelet activation may also affect microvesiculation in stored platelets. A current study has indicated that upon platelet activation, cytosolic phospholipase A2 (PLA2) activity with subsequent AA production can result in dimerization of 14–3-3ζ and its interaction with the cytoplasmic domains of GPIbα in lipid rafts. While this interaction induces the stabilization and clustering of GPIbα, on the other hand, it causes the loss of the membrane anchoring interaction of Filamin A with this receptor, which affects platelet surface integrity and makes the membrane prone to microvesiculation [43].

### Ectodomain shedding in platelets

Ectodomain shedding through limited proteolysis is a general mechanism to modulate the function of surface transmembrane proteins. This process usually occurs during cell activation and is associated with the proteolysis of a broad range of transmembrane macromolecules including adhesion receptors, numerous cytokines, growth factors and their receptors. However, resting (non-stimulated) cells also experience a basal level of constitutive ectodomain shedding which is increased in a time-dependent manner, while can be dramatically enhanced in response to activating stimuli or apoptotic conditions [44, 45]. Platelet adhesion and activation has been shown to be associated with surface protein shedding [46] and since platelets have not the translational capability to renew their full complement of surface proteins, ectodomain shedding is considered to be a rapid and irreversible key mechanism which effectively down-regulates the surface expression of platelet receptors and their signaling capacities [11, 13]. Endogenous platelet metalloproteinases (sheddases) including a disintegrin and metalloproteases (ADAMs) and matrix metalloproteinases (MMPs) are the key players in platelet ectodomain shedding [47]. Ectodomain shedding has been reported as an important mechanism for the regulation of GPIbα and GPV expressions on the platelet surface. In addition to down-regulation of the interaction between GPIb-V-IX and vWF, this phenomenon has been also suggested to be responsible for the clearance of aged platelets from the circulation [48]. Actually desialylation of GPIb-V-IX through sialidase activity also causes platelet clearance while inducing the irreversible formation of receptor clustering and the ectodomain shedding of GPIbα and GPV from the platelets [49, 50]. ADAM17 is the most prominent endogenous sheddas, which cleaves both GPIbα and GPV at specific sites releasing a 130- and an 80-kDa soluble fragment, respectively [48]. The 130 kDa fragment is referred as glycocalicin (GC) and circulates in plasma at a concentration of 1 to 3 μg/mL [51] . Generally, remarkable function of ADAM 17 occurs during platelet activation by a variety of agonists, however due to the presence of constitutive levels of mature ADAM17, even resting platelets experience basal levels of GPIbα and GPV shedding. Evidence for the physiological regulation of GPIbα by ADAM 17 can be derived from the observation that the concentration of glycocalicin in the plasma of the mice deficient in functional ADAM17 is considerably lower than wild type animals [52]. In addition to cell activation, GPIbα shedding also occurs during cell death. Treatment of platelets with carbonyl cyanide 3-chlorophenylhydrazone (CCCP) provokes mitochondrial injury and platelet apoptosis similar to that observed during the storage of platelets in vitro. In both scenarios, ADAM17 has been suggested as the responsible protease [45, 52, 53]. As the main collagen receptor, GPVI is another molecule which is shed from the platelet surface during platelet activation by agonists as well as shear stress. However, these shedding events are mainly modulated by ADAM10 while leading to the release of a soluble ~ 55-kDa fragment with a ~ 10 kDa platelet-associated remnant fragment. In addition, similar to GPIbα, the mitochondrial damage also shown to be associated with GPVI shedding in a mechanism which is mainly ADAM 17 dependent [54]. Resting platelets also constitutively present both ADAM 10 and ADAM 17. However contrary to what already mentioned for GPIb and despite the presence of ADAM10 with detectable proteolytic activity on the surface of resting platelets, GPVI expression has shown to be quite stable on circulating inactivated platelets. This might be due to cryptic nature of vulnerable site of GPVI which only expose to ADAM 10 proteolytic activity under the condition of ligand binding or membrane perturbation in elevated fluid shear stress [55].

#### Mechanistic features for the shedding of platelet adhesion receptors

Several lines of evidence suggested that ADAM-dependent shedding of platelet adhesion receptors can be modulated by Ca^2+^ elevation, protein kinase C (PKC) activation, PS exposure, and caspase activity [56]. A calcium-modulated protein, calmodulin is one of the important modulators of ectodomain shedding. It has been shown that the direct attachment of calmodulin to juxtamembrane cytoplasmic sequences of GPV, GPIbβ (from GPIb-V-IX complex) and GPVI receptor can interfere with the activity of the sheddase. This is supported by the observation that dissociation of calmodulin from the ectodomain cleavage sites, induced by calmodulin inhibitors such as W7, renders receptors more vulnerable to shedding. It has been shown that calmodulin disassociation from these inhibitors significantly increases the levels of sGPVI and GC. On the other hand, signaling events followed by platelet activation can increase intracellular calcium while its interaction with calmodulin can be associated with rapid conformational changes of the molecule and its dissociation from receptor cleavage sites which make them vulnerable to the proteolytic activities by ADAMs [57]. The another possibility is that the interaction between those affected calmodulin and some cytoskeleton modulators such as myosin light chain kinase (MLCK) causes cytoskeletal rearrangement which may induce shedding event by the effects on either receptors stability or ADAMs activity [40, 57, 58]. Additionally, calmodulin may affect receptor shedding through its involvement in other pathways including calcium/calmodulin-dependent protein kinase type I/II (CaMKI/II) while calmodulin antagonists also showed to induce receptor shedding by the activation of apoptotic pathways or calcium elevation [59, 60]. Generally, platelet activation and Ca^2+^ elevation is associated with increased levels of intracellular reactive oxygen species (ROS) [61]. ROS–induced oxidation of cysteine residues located on cysteine-rich domain of ADAMs can activate these proteolytic molecules, while ROS interaction with intracellular cytoplasmic domains of ADAMs may also increase their affinity with their substrate [58]. On the other hand, the direct or indirect interaction of ROS with cytoplasmic domains of the adhesive receptors GPVI may also induce shedding events. It has been indicated that following ligand binding of GPVI, rapid oxidation of an unpaired thiol in the cytoplasmic tail of this receptor results in the formation of GPVI homodimers, preceding down-stream signaling and ectodomain metalloproteolysis of GPVI [62, 63]. ROS also can indirectly affect the receptors and induce their shedding by the oxidation of cysteine residues of different related protein kinases including p38-MAPK (mitogen-activated protein kinase), PTP (protein tyrosine phosphatase), SFK (sarcoma family kinase) and PKs C, G, or A [64–66]. It is known that ADAM17 (as the main sheddas of platelet GPIbα and GPV) is activated via a p38 MAPK-dependent pathway which is per se modulated by accumulating ROS [67]. On the other hand, activated p38-MAPK can lead to the dimerization of the protein 14,3,3ζ attached to the cytoplasmic domain of GPIbα. This induces the receptor clustering and its disassociation from filamin which makes it unstable and susceptible to shedding events [40]. ROS-induced activation of p38-MAPK, PKC and SFK can enhance NADPH oxidase activity leading to further ROS generation [68]. The generated ROS can activate other signaling pathways such as PLC and PI3K which increase intracellular calcium by its either release from platelet DTS or entrance by specific channels [61]. Consequently, this ROS-induced calcium influx can enhance receptor shedding in the way already discussed.

## Proteolytic modulation of platelet receptor integrin α_IIb_β_3_

It has been currently shown that platelets can experience the ectodimain shedding of α_IIb_β_3_ under non-physiological shear stress while the concentration of this receptor components has been increased in microparticle-free plasma [69]. However, more studies are required to characterize a specific sheddase for α_IIb_β_3_ or define an in vivo pathophysiologic importance for this observation and its relevance to the mechanisms of integrin down-regulation. Additionally, a recent study also claimed that oxidative stress can induced modulation of platelet integrin α_IIb_β_3_ expression and shedding in patients implanted with continuous flow left ventricular assist devices (CF-LVADs). However, in this study samples examined for the receptor shedding analysis were not microparticle-free and since PMPs are rich of α_IIb_β_3_, both observed reduction in the expression of α_IIb_β_3_ and higher levels of soluble integrin could be resulted from platelet microvesiculation rather than ectodomain shedding [70]. Regardless of these reports, so far all other studies have not reported ectodomain shedding for α_IIb_β_3_ in any in vivo or in vitro experimental models. On the other hand, In different condition of either agonist-induced or storage-dependent PMPs formations, our western blot analysis also confirmed that using multi-steps ultra-centrifugation can totally reduce the soluble levels of α_IIb_β_3_ in microparticle-free samples [54] . However, studies showed that, following platelet activation especially within sustained calcium influx, calpain-dependent proteolytic cleavage of cytoplasmic part of β3 may affect α_IIb_β_3_ activation and clustering [48] .Several line of evidence have also reported calpain-mediated cleavage of integrin-dependent signaling molecules (including FAK, Src, PTP-1B) and some related cytoskeletal proteins (such as talin, filamin-1, and cortactin). This may also be considered as an alternative proteolytic pathway which can control the adhesive function of α_IIb_β_3_ while regulating thrombus formation. However, the role of calpain in regulating integrin activity still remains controversial as shown that this protease may present either positive or negative effects on function of α_IIb_β_3_ platelets [71].

## Platelet receptors crosstalk and ectodomain shedding

Receptors crosstalk is also considered as another feature of shedding mechanism. The studies showed that the GPIb ligation causes its cross-interaction with FC receptors (FCR) γ and FCRIIa through the PI3K signaling pathway [72]. The interactions induce signaling cascades down-streams of these two receptors leading to the enhancement of GPVI shedding [73]. C-type lectin-like receptor 2 (CLEC-2) is a currently described molecules expressed by platelets, which can interact with Podoplanin as a widely expressed molecule outside of the vasculature while inducing activating signals through a hemimmunoreceptor tyrosine-based activation motif (hemITAM). It has been shown that the ligation of this receptor is associated with the proteolytic cleavage of GPVI and FcgRIIa. However, there is no evidence that shows CLEC-2 shedding during platelet activation [31] .Integrin involvement in ADAMs activation is another calcium-dependent mechanism which can control shedding events. It is known that followed by inside-out signaling, integrin gets primed while its ligation with fibrinogen can induce significant activating outside-in signals which ignite calcium influx, ROS generation and consequent receptors shedding [61, 74]. Still, there is one more intriguing mechanism by which integrin can modulate shedding through its direct interaction with ADAMs. In one side, integrin binding to the ADAMs can activate these molecules enhancing the shedding of adhesive receptors while this interaction also can modulate integrin ligation capacities attenuating outside-in signaling [75]. It seems that, this mechanism can efficiently down-regulate platelets adhesive function through the either decrease in platelet activation or increase of receptor shedding. On Receptor shedding could also be essentially Ca^2+^ independent. There are some PKC mimetic compounds, such as phorbol-myristate-acetat (PMA) which act independent of Ca^2+^ while significantly increasing ADAMs activities and shedding [56, 76].

## Platelet apoptosis and receptor shedding

Platelet apoptosis is associated with the significant ectodomain shedding of surface receptors. Ex vivo studies showed that the treatment of human platelets with BH3 mimetic agents can decrease platelet adhesive function due to ectodomain shedding of GPIbα and GPVI. On the other hand, thrombocytopenic mice deficient in Bcl-xL also showed the reduced expression of GPIbα and GPVI in platelets [46, 77], confirming the important role of apoptotic pathway in shedding process. In addition to BH3 mimetic agents that induce a time- and dose-dependent decrease in platelet adhesive function resulted from ectodomain shedding of GPIbα and GPVI, the mitochondrial poison carbonyl cyanide 3-chlorophenylhydrazone (CCCP) also creates premature platelet ageing and shedding of these platelet receptors [78]. Although, apoptosis-induced shedding of receptors is mainly independent of Ca2+, its exact mechanism is not well characterized yet, as BH3 mimetic agents like ABT 737 also showed to induce a small increases in Ca2+ in a minor platelet population [79]. It has been shown that under apoptotic condition, metalloproteinase inhibitors can significantly reduce receptor shedding. Therefore, it is postulated that the activation of metalloproteinase downstream of mitochondrial injury and caspase activation can be the main event that regulates receptor shedding under apoptotic condition. Studies showed that target receptor shedding by activated ADAM17 is modulated in a p38-dependent manner while the inhibition of p38-MAPK can block the shedding of GPIbα in stored platelets [46]. On the other hand, studies also confirmed that p38-MAPK significantly plays a role in ABT 737-induced platelet apoptosis; however whether the activation of this kinase during apoptosis can be also involved in platelet receptor shedding requires further investigation [80]. Of note, in apoptotic condition, the possible mechanism by which MAP kinase regulating ADAMs activity and shedding cannot be ROS-dependent as already described under activation status because BH3 mimetic agents did not show to whether induce or increase intra platelet ROS generation [80].

## Physiological significance for the shedding of platelet adhesive receptors

In a physiological basis, shedding of adhesion receptors is considered as part of a mechanistic process facilitating platelet spreading via the sequential events of platelet de-attachment and re-attachment at the tips of filopodia or the leading edge of lamellipodia. In addition receptor shedding is an important mechanism modulating thrombotic function through a controlled down-regulation of platelets reactivity [48]. However, in some cases, the uncontrolled shedding of adhesion receptors can molest platelet function and hemostasis [81]. Lowered surface density of GPIbα and GPVI due to the shedding can attenuate signaling capacity which affects platelet activation and secretion. This is in addition to the platelet adhesive malfunction which is in a direct correlation with lower expression of these receptors. On the other hand, ectodomain shedding of adhesion receptors can also decrease the number of platelet-matrix contacts, which potentiates thrombo-embolic events [48] . It has been shown that in thrombi formed ex vivo, pro-coagulant platelets undergoing ADAM-induced glycoprotein shedding are less-adhesive due to defects in GPIbα and GPVI dependent adhesion to VWF, collagen, and fibrin. In addition calpain-induced integrin cleavage in pro-coagulant platelets can be also considered as another reason aggravating platelet signaling capacity and adhesive capabilities [56]. Notably, pro-coagulant function is associated with the excessive microparticle formation leading to the loss of platelet membrane containing adhesion receptors [81]. Thus in a same scenario but with a different mechanism, platelets lose more adhesive capacities within post activation stage.

## Platelet storage and increasing loss of adhesion receptors

Transfusion of platelet concentrates (PCs) is one of the most important therapeutic approaches in the management of thrombocytopenia and bleeding complications. Regardless of preparation techniques, stored platelets gradually experience inevitable deleterious changes so-called platelet storage lesion (PSL) that may lead to a progressive structural and functional damage. These changes which begin from the time of platelets isolation till the transfusion to a recipient are mainly induced by either reversible or irreversible increases in the basal levels of platelet activation. The most important phase of PSL is associated with irreversible changes in platelet morphology and function, which are significantly initiated with platelet α granules release and its conversion from pro-aggregatory status to pro-inflammatory phenotypes identified by the expression of P-selectin and CD40L [54] . It has been shown that along with platelet activation, the increased levels of intracellular calcium acts as the main modulator of signaling events which results in platelet receptor ectodomain shedding and membrane loss due to microparticulation. Both of these phenomena cause progressive loss of adhesive receptors during platelet storage [82]. Notably this extensive loss of platelet receptors may affect its proper function required for therapeutic uses [54, 83]. Decreasing levels of GPIbα expression during the storage of platelet concentrates (PCs) were reported by several studies [54]. Receptor ectodomain shedding and platelet membrane loss due to microparticulation are two important causes for this gradual decrease [82]. We already indicated that the continuous shedding can lead to significant decrease in GPIbα expression in 5-day stored platelets [54]. It is postulated that the down-regulation of GPIbα expression affect the adhesive function of transfused platelet in elevated arterial shear rates [84]. Obviously the older platelet products with the lower GPIbα expression may show less functionality after transfusion. This is in addition to the fact that metalloproteinase- dependent loss of surface GPIbα plays a role in the clearance of aged platelets from the circulation [52]. Therefore, the higher levels of shedding for this receptor in older PCs could be also associated with a rapid clearance of transfused platelets leading to reduced platelet recovery and survival. GPVI expression also showed to be modulated during storage. We already demonstrated a decreasing expression of GPVI during storage where was directly correlated with the GPIbα expression. A negative correlation between GPVI expression and shedding was also observed, a finding that verifies the main role of ectodomain shedding in the modulation of GPVI expression. It is worthy to know that given the absence of GPVI in the proteome of platelet microparticles, dislike GPIbα, the reducing levels of GPVI expression should be mainly due to ectodomain shedding rather than microvesiculation. A direct correlation between the shedding levels of P-selectin and GPVI during storage suggested this adhesion receptor as a valid marker of PSL [54] . This finding was also consistent with data from clinical studies in which the levels of soluble GPVI was correlated with soluble P-selectin in patients with acute coronary syndrome and or with Acute Ischemic Stroke [85]. Further studies also suggested soluble GPVI as a helpful biomarker to monitor platelet activation during anti-platelet therapy [86]. Notably considerable shedding of GPVI and its association with decreasing expression of this receptor on the surface of stored platelets can affect GPVI dependent platelet function during storage. Some studies showed significant attenuation in platelet adhesion capacity to collagen in stored PCs [87, 88]. We also showed a direct correlation between this observed decreasing adhesive capacities with the increasing levels of GPVI shedding during storage. However, whether the observed loss of GPVI during storage can render platelet signaling capacities and integrin activation was another important question which has been addressed by the observation of attenuating platelet aggregation in responses to collagen during storage and its significant reverse correlation with GPVI shedding in stored PCs [89] .

## Clinical significance of PSL

Although clinical research has not yet found any significant correlation between PSL and post-transfusion adverse effects or bleeding events in the patients, these studies have confirmed that storage duration can affect platelets survival in circulation. It is known that transfusion of fresher platelets is accompanied with higher CCI (corrected count increment) and more prolonged transfusion intervals in patients [90]. In other word, clinically, transfusion of the longer stored PCs has been associated with reduced CCI, platelet recovery and survival [91]. This might be due to the fact that in longer stored products, platelets with P-selectin exposure, GPIbα shedding and reduced membrane-associated sialic acid are selectively cleared from circulation [87, 92] .Using the mice models, in vivo studies showed that in stored platelets the inhibition of GPIbα shedding by metalloproteinase inhibitor or 5G6 Fab fragment can promote post transfusion recovery of platelets while improving their hemostatic function [87, 93]. Conclusively these studies suggest that GPIbα shedding reduces platelet reactivity while increasing its clearance in vivo. However, while lower CCI, platelet recovery, clearance and survival are much related to expression levels of adhesion receptors has not well defied yet.

## Conclusion

Therapeutic platelet transfusion can be complicated by platelet refractoriness either modulated by immune or non-immune causes. Immune-related refractoriness especially those induced by antibodies against HLA antigens are the most studied causes of post transfusion platelet malfunctions. However platelet storage dependent lesion seems to play an important role in the premature clearance of transfused circulating platelets, which also results in lower efficacy for platelet transfusion. Evidently platelet storage is associated with the ectodomain shedding and microvesiculation which down-regulate platelet adhesion receptors, GPIbα and GPVI. This is in addition to the storage-dependent loss of integrin activity, which is also crucially involved in platelet aggregability and thrombus formation. Whereas these alterations have shown to irreversibly affect platelet function in vitro, clinical observations have indicated that the premature clearance or lower survivals rather than platelet malfunction is the only important adverse effect of platelet transfusion. Therefore given the improvement of hemostasis in the patients who transfused the longer-stored platelets, some studies suggested that platelets dysfunction due to PSL is generally restored after transfusion. However, this is a controversial viewpoint, as basic studies have confirmed that platelet adhesion during storage is affected by irreversible down-regulating mechanisms such as ectodomain shedding, micopaticulation and other proteolytic cleavage which permanently destroy some receptor adhesive and signaling function. Thereby highlighting detailed mechanisms involved in down-regulation of platelet adhesion receptor,This review may benefit the readership to better judge whether the functional capabilities of stored platelets can be properly recovered after transfusion or not.

## Data Availability

Data sharing not applicable to this article as no datasets were generated or analysed during the current study.

## Abbreviations

ADAMs: A disintegrin and metalloproteases
AIDS: Acquired immune deficiency syndrome
CaMKI/II: Calcium/calmodulin-dependent protein kinase type I/II
CCCP: Carbonyl cyanide 3-chlorophenylhydrazone
CCI: Corrected count increment
CD40L: CD40 ligand
CF-LVADs: Continuous flow left ventricular assist devices
CLEC-2: C-type lectin-like receptor 2
FCR: FC receptors
GC: Glycocalicin
GPIbα: Glycoprotein Ibα
GPVI: Glycoprotein VI
hemITAM: Hemimmunoreceptor tyrosine-based activation motif
LAMP3: Lysosomal-associated membrane protein 3
MAC-1: Machrophage-1 antigen
MAPK: Mitogen-activated protein kinase
MMPs: Matrix metalloproteinases
MPs: Microparticles
MLCK: Myosin light chain kinase
NETs: Neutrophil extracellular traps
OCS: Open canalicular system
PI3K: Phosphoinositide 3-kinases
PMA: Phorbol-myristate-acetat
PS: Phosphatidylserine
PLA2: Phospholipase A2
PAF: Platelet activating factor
PCs: Platelet concentrates
PECAM: Platelet endothelial cell adhesion molecule
PMPs: Platelet derived microparticles
PSL: Platelet storage lesion
PKC: Protein kinase C
PSGL-1: P-selectin glycoprotein ligand-1
PTP: Protein tyrosine phosphatase
ROS: Reactive oxygen species
SFK: Sarcoma family kinase
TF: Tissue factor
vWF: Von Willebrand factor
